# A New Mode of Treating Puerperal Fever

**Published:** 1873-01

**Authors:** Charles Bell


### A NEW MODE OF TREATING PUERPERAL FEVER.

BY DR. CHARLES BELL.

It is not my intention to enter on a lengthened dissertation on puerperal fever, as it is fully described in many of our works on midwifery, and my object is merely to bring more prominently under the notice of the Society a mode of treatment which I have been induced to recommend, from its successful results in diseases of a similar nature, as well as in the few cases of this disease in which I have had the opportunity of employing it. For, although

I have been connected with the Royal Maternity Hospital for many years, only one case of puerperal fever has occurred during the period I was doing duty. I ascribe this immunity from the disease to the liberal use of the permanganate of potash, both as a lotion and in the form of a liniment, in making examination per vaginam, and to a strict attention to ventilation, and to the avoidance of over-crowding the wards. I feel satisfied that there is greater risk to the parturient woman from a close contaminated atmosphere, than from the free admission of pure air into the lying-in chamber. I have, therefore, always ordered one of the windows of the wards to be kept partially open.

The more we study puerperal fever, the correctness of the opinion entertained by Sennertus and Riverius will appear the more obvious, namely, that it is a blood disease arising from the absorption of a virulent poison into the system, which is communicable from one individual to another, and may also be inhaled from the atmosphere. This circumstance was fully illustrated by M. Peu, in his description of the epidemic fever which appeared in the Hotel Dieu in 1664, when a great number of women died, in consequence of the number of wounded persons who were accommodated under the lying-in ward. This was the first appearance of the disease in the epidemic form, and it was peculiar in its character, as it was preceded by hemorrhage, and was attended by numerous internal abscesses. It formed a strong contrast to the next epidemic, which occurred in Paris in 1746, and proved fatal to many women, both in the hospital and to those who were delivered in their own houses. According to Montonu, it "commenced with diarrhœa; the uterus became dry, hard and painful; it was swollen, and the lochia had not their ordinary course; the women experienced pain in the bowels, particularly in the situation of the broad ligaments; the abdomen was tense; and these symptoms were sometimes joined with pain in the head and cough. On the third or fourth day after delivery, the mammæ became flaccid. On opening the bodies, matter like curdled milk was found on the surface of the intestines; a milky serous fluid in the hypogastrium; a similar fluid was found in the thorax of certain women; and when the lungs were divided they discharged a milky putrid lymph. The stomach, intestines and uterus appear to have been inflamed."

It may not be uninteresting to refer to the character of the disease as it appeared in the epidemic which occurred in Paris and Lyons in 1750, as illustrating still further the complications which attend the disease, and prove still more its zymotic character. Poteau informs us that the uterus was found enlarged, and its internal surface soft and black, and its parietes were livid and gangrenous.

While puerperal fever may be complicated in the manner just described, it is not a necessary consequence, as, like small-pox in its most fatal form, it runs its course so rapidly in some cases that there is no time to form any local lesion which can be ascertained during life or discovered after death. Dr. Rigby informs us that, in some of the cases which came under his notice in the general lying-in hospital, there was "neither time nor power sufficient to produce either symptom or trace of inflammation, the powers of life having from the commencement sunk under the deadly influence of the disease." He therefore adds, "that, of all the diseases to which the lying-in woman is exposed, puerperal fever is the most to be dreaded, and that there is none in which the accoucheur is frequently more helpless."

In a disease of such virulence, it is of the utmost importance that a remedy should be discovered to moderate its severity. I am convinced of its similarity to erysipelas—a circumstance which has been proved, not only by its appearing epidemically along with that disease, as happened in Barnsley in 1808, and in Leeds in 1809, but there are instances on record* in which puerperal fever has been induced by those attending patients suffering from eyrsipelas, and then attending women in their confinement; and there have also been instances of puerperal fever patients producing erysipelas in their nurses. I have also observed both diseases existing at the same time in the same individual. I was therefore induced, many years ago, to suggest that the treatment which I had found so beneficial in erysipelas should be adopted in puerperal fever. I had no opportunity, however, of putting it in practice until a comparatively recent period, when I employed it in the only case which came under my care in the Maternity Hospital, and although the case was very severe, and the patient's life was despaired of, it proved successful, and the woman left the hospital in health. I have already reported this case to the Society. The success which attended this case led me to adopt it in another case, which I was requested to see by Professor Simpson, which was considered hopeless, yet the patient left the hospital in perfect health. Through the kindness of Dr. Young, I had an opportunity of employing the treatment in a patient of his, whom I attended in a premature labor. She was seized with puerperal fever soon after her confinement, and I despaired of her recovery, but she was ultimately restored to health, I understand, for I ceased my attendance before she was quite recovered.

I recommend small doses of calomel and James's powder in proportion of a twelfth of a grain each, along with a grain of white sugar, carefully mixed and given regularly every two hours until the bowels are freely moved; and thirty drops of the muriated tincture of iron every three hours. The vagina to be washed out several times a day with Condy's red fluid and tepid water, and a linseed poultice applied to the abdomen. If this treatment is regularly and fully carried out, and not in the *timid*, *partial* way which many practitioners do in erysipelas, and then undervalue the treatment, I feel certain that it will give the best chance of cure to the patients.

Dr. James Young believed that great benefit was obtained from using calomel in the manner described by Dr. Bell; he had lately suffered from an attack of erysipelas, and been treated with the tincture of the muriate of iron and calomel, in doses of a twelth of a grain, with the best results. He had never seen head symptoms in puerperal fever, and he looked upon diarrhœa as one of the most serious complications you could have in that disease.

Dr. Ritchie had tried calomel as a purgative, in the Children's Hospital, in doses of a twelfth of a grain, but without any effect. He considered it important to distinguish the special character of the fever; in a fever of the typhoid type, he would not be inclined to use the tincture of the muriate of iron.

Dr. Bell regarded puerperal fever as a disease *sui generis*, and that you may have complications occurring in it as in any other disease. The tincture of the muriate of iron, he believed, lowered both the pulse and temperature. The efficacy of the powder depends greatly on the manner in which it is compounded, and the regularity with which it is given. When these have been properly attended to, he has never found it to fail to move the bowels, and to diminish fever; but if they are neglected, it is not surprising that it should cause disappointment.—*American Journal Obstetrics.*

